# Protein Set Transformer: A protein-based genome language model to power high diversity viromics

**DOI:** 10.21203/rs.3.rs-4844047/v1

**Published:** 2024-09-23

**Authors:** Cody Martin, Anthony Gitter, Karthik Anantharaman

**Affiliations:** 1 Department of Bacteriology, University of Wisconsin-Madison, Madison, WI, USA; 2 Microbiology Doctoral Training Program, University of Wisconsin-Madison, Madison, WI, USA; 3 Department of Biostatistics and Medical Informatics, University of Wisconsin-Madison, Madison, WI, USA; 4 Morgridge Institute for Research, Madison, WI, USA; 5 Department of Computer Sciences, University of Wisconsin-Madison, Madison, WI, USA; 6 Department of Integrative Biology, University of Wisconsin-Madison, Madison, WI, USA

**Keywords:** viromics, viral metagenomics, genome language model, protein language model, transformer

## Abstract

Exponential increases in microbial and viral genomic data demand transformational advances in scalable, generalizable frameworks for their interpretation. Standard homology-based functional analyses are hindered by the rapid divergence of microbial and especially viral genomes and proteins that significantly decreases the volume of usable data. Here, we present Protein Set Transformer (PST), a protein-based genome language model that models genomes as sets of proteins without considering sparsely available functional labels. Trained on >100k viruses, PST outperformed other homology- and language model-based approaches for relating viral genomes based on shared protein content. Further, PST demonstrated protein structural and functional awareness by clustering capsid-fold-containing proteins with known capsid proteins and uniquely clustering late gene proteins within related viruses. Our data establish PST as a valuable method for diverse viral genomics, ecology, and evolutionary applications. We posit that the PST framework can be a foundation model for microbial genomics when trained on suitable data.

## Introduction

Viruses are the most abundant biological entity on the planet and inhabit every ecosystem. Understanding how viruses modulate microbiome community dynamics and functional outputs is an active area of research that spans various scales from global biogeochemistry^1^ to human health and disease^2^. Despite the sheer abundance and influence of viruses, comprehensive large-scale viral metagenomics (viromics) studies are severely hindered by the enormous genetic diversity of viruses as most genomics tools rely on sequence similarity to existing reference databases. These problems are compounded by the lack of universal genes in viruses, complicating phylogenetic and comparative analyses across diverse groups of viruses. Overall, these challenges have impeded the development of viromics software that is both accurate and scalable to increasingly diverse viral datasets. Thus, there is a clear need to develop data-driven frameworks to study viruses at-scale using generalizable genomic principles instead of simple sequence homology-based methods.

Protein language models (pLMs) are promising deep learning frameworks for generalizable genomics. Trained on corpuses of millions of proteins^3–5^, pLMs have been shown to model amino acids patterns in protein sequences akin to reading words in sentences, capturing biochemical, functional, and structural features of proteins using contextual information of the amino acids within a protein^4,5^. Applications of pLMs to viral datasets have demonstrated increased capacity for protein function annotation^6,7^ and host prediction^8^. However, these studies only focused on specific tasks without considering that pLMs could be universally beneficial for a variety of viromics tasks^9^, thus missing out on the true potential of foundation pLMs. An additional shortcoming of the pLMs themselves is that they do not account for evolution-driven genome organization. Recent work has addressed this issue by contextualizing pLM embeddings across short genomic fragments^10^ and even representing the entire genome as an aggregation of the pLM embeddings^8^. However, each of these models only targets one specific kind of representation: the former represents proteins with added genome context, while the latter represents genomes as a weighted sum of protein embeddings subject to a specific classification task. Thus, none of these approaches are truly generalizable to a variety of viromics tasks that require both protein- and genome-level reasoning.

Here, we present our Protein Set Transformer (PST), a protein-based genome language model that uses an encoder-decoder paradigm to simultaneously produce both genome-contextualized protein embeddings and genome-level embeddings within a single end-to-end model. We pretrained a foundation viral PST (vPST) model on >100k high-quality dereplicated viral genomes encoding >6M proteins and evaluated on a distinct test dataset of >150k high-quality viral genomes encoding >7M proteins from IMG/VR v4^11^. We demonstrate that vPST better relates viral genome-genome relationships based on shared protein content. Further, we observe that only vPST can consistently cluster operationally related proteins like late gene proteins, indicating the importance of genome context-aware training. Additionally, vPST protein embeddings are associated with protein structure relationships, as demonstrated by clustering capsid-fold-containing proteins with no annotated function with annotated capsid proteins.

Notably, neither the genome-contextualized vPST protein embeddings nor the genome embeddings were learned with respect to any external labels, meaning that they will be useful for a wide range of applications. Due to this flexibility of the vPST, we propose that the vPST can be used for transfer learning to model other viral-centric tasks such as viral gene and genome identification, genome quality control, genome binning, taxonomy, and host prediction, which are major components of viromics research^9^. Thus, we expect that the vPST will be foundational to future viromics studies. Further, we posit that the PST architecture can be a general-purpose model for microbial genomics when trained on microbial instead of or in addition to viral genomes.

## Results

### Developing the Protein Set Transformer (PST) as a genome language model

**Figure 1.**
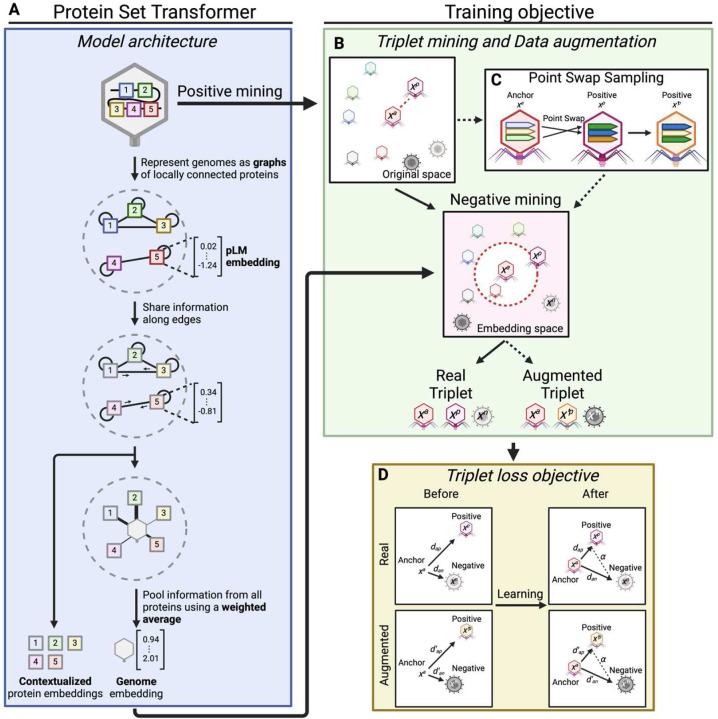
The Protein Set Transformer (PST) architecture and training regime. **A**) General overview of the graph-based PST for learning genome representations from contextualized protein embeddings. Each protein is represented by an ESM2 protein embedding. The PST internally represents each genome as a graph, consisting of multiple subgraphs of fully connected, locally adjacent proteins. The size of each subgraph is a tuned hyperparameter. The PST uses multi-head attention both to contextualize protein embeddings within each genome and to learn per-protein weights for a weighted averaged over each genome. See Extended Data Fig. 1 for a modeling-centric view of the PST. Both protein and genome representations can be used for the appropriate downstream task. **B**) Triplet mining workflow that includes the data augmentation technique **C**) PointSwap sampling. For each training genome, a positive genome is identified from the ESM2 embedding space defined as the minimum Chamfer distance. Then, a negative, less related, genome is chosen from the PST embedding space that is the next farther genome after the positive. We augment our training data by creating hybrid genomes that swap similar protein vectors between each genome and its positive genome. **D**) Pictorial representation of the triplet loss objective function used to train the viral PST (vPST). The operational objective of triplet loss is to embed each genome and its positive genome closer in embedding space than each genome and its negative genome, within a tunable distance margin.

The PST (Fig. 1A, Extended Data Fig. 1) models genomes as sets of proteins using principles from the natural language processing and set^12^ and pointset^13^ transformer^14^ fields. We thus refer to the PST as a protein-based genome language model, since it contextualizes protein information at genome-scale. In PST, all proteins from each genome are embedded using the well-established ESM2 pLM^3,4^. Unlike the Set Transformer^12^, the PST concatenates small vectors onto the pLM embeddings to model both the protein genome position and coding strand. This set of updated protein embeddings from each genome are fed to the PST encoder, which uses multi-head attention^14^ to contextualize the protein representations within each genome (referred to as simply “PST protein embeddings” from here out). These PST protein embeddings can be used for protein-level tasks like protein classification and functional annotation. In the end-to-end PST, the PST protein embeddings are further passed to the PST decoder, which also uses multi-head attention to weigh the relative importance of each protein in a genome. These weights are used for a weighted average of the contextualized proteins to produce a genome representation.

A common training objective for language models that is used by a similar protein-based genome language model^10^ is masked language modeling^15^, which involves predicting masked tokens (words) in sentences from the rest of the sentence. In the case of genome sentences composed of protein words represented as dense vectors, masked language modeling is less intuitive and likely overcomplicates training. We instead opt to mirror relationship-guided genomics to better understand patterns of genetic diversity using the triplet loss function^13,16^ (Fig. 1B, 1D). During self-supervised pretraining of the vPST foundation model, triplet loss uses the distance in vPST embedding space as a measure of genome-genome relatedness. In vPST, genome-genome relationships are implicitly conditioned on protein-protein relatedness. Briefly, triplet loss involves the formation of genome triplets, consisting of one as an anchor, the genome most related to the anchor as a positive example, and a genome less similar than the positive genome as a negative example^13,16^ (Fig. 1B, 1D). Positive examples are defined using the Chamfer distance in the input embedding space among genomes within a training minibatch, while negative examples are sampled in the vPST embedding space. Chamfer distance is computed as the average minimum of protein-protein distances for pairs of genomes, meaning that the positive genome has the most similar proteins to the anchor genome. The objective of triplet loss is to embed the positive genome closer to the anchor than the negative within a tunable margin (Fig. 1D).

To help the vPST learn more generalizable representations, we used the data augmentation technique PointSwap^13^ (Fig. 1C) for each genome and its most related genome defined by Chamfer distance above (Fig. 1B). Each genome pair swaps protein vectors that are most similar at a defined, tunable rate, analogous to homologous recombination. We then update the triplet loss objective to include maximizing the similarity between the anchor genome and its corresponding augmented hybrid genome produced by PointSwap.

### Tuning the vPST using a modified Leave-One-Taxon-Out cross validation strategy

To train the vPST viral foundation model, we collected 103,589 viruses from 12 different publicly available sources^1,17–27^ as a training dataset (Extended Data Fig. 2B). 151,255 viruses from IMG/VR v4^11^ that were distinct at the nucleotide level (<95% average nucleotide identity over 85% of either genome) from the training viruses were used as the test dataset. Dereplication at the nucleotide level was sufficient to reduce train-test genome similarity at the protein level (Extended Data Fig. 2A). Most viruses in either set were predicted to encode between 2–100 proteins (Extended Data Fig. 2C) and to be Duplodnaviria, Monodnaviria, or Riboviria (Extended Data Fig. 2D). Additionally, most were from environmental sources not associated with a holobiont (Extended Data Fig. 2D), especially marine systems. Among the viruses with a known or predictable host, most are bacterial viruses (Extended Data Fig. 2D).

We tuned 2 different vPSTs with small (6-layer, 8M param) and large (30-layer, 150M param) ESM2 protein embeddings, respectively, using a variant of leave-one-group-out cross validation (CV), where the group is the viral taxonomic realm. In our variation, the Duplodnaviria group is always included as a CV training fold since this group composes a significant fraction of our training set (65.4%, Extended Data Fig. 2D). This CV setup notably helps choosing model hyperparameters optimal for all viruses rather than just the most abundant. The best models were chosen based on the lowest triplet loss averaged among all folds at the end of tuning (Extended Data. 3AB). Using this strategy, we tuned training-specific (dropout, layer dropout, learning rate, weight decay, batch size), model-specific (number of attention heads and encoder layers), PST-specific (chunk size), PointSwap-specific (rate), and triplet loss-specific (distance margin, scale factor) hyperparameters (Extended Data Fig. 3CD). For the small vPST, fewer attention heads and encoder layers led to optimal performance, while the reverse is true for the large vPST, likely reflecting the increased information capacity of larger pLM embeddings. Increasing values of the AdamW optimizer (PyTorch v2.0.0) weight decay parameter, increasing values of the PointSwap rate, and decreasing values of the triplet loss distance margin led to decreased loss (better performance) for both vPST sizes. After hyperparameter tuning, we trained a final model for each ESM2 input using the best hyperparameters (Extended Data Fig. 3E). The remaining results are based on these 2 models that we refer to as “pst-small” (5M parameters) and “pst-large” (178M parameters), respectively, when discussing both the learned genome and protein representations.

### The vPST captures biologically relevant information about viral genomes

**Figure 2.**
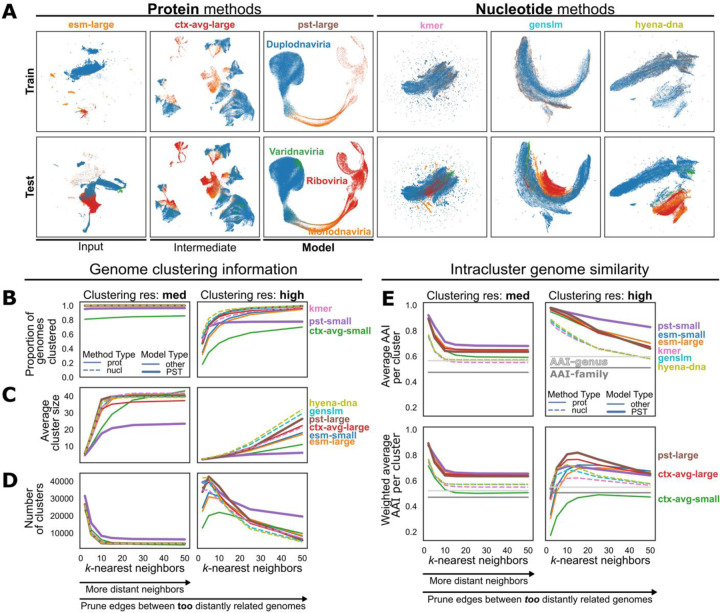
The vPST learns biologically meaningful genome representations for diverse sets of viruses. **A**) UMAP dimensionality reduction plots for the genome embeddings produced by each method, color coded by the viral realm. “Kmer” represents 4-mer nucleotide frequency vectors. “Ctx-avg” methods are averages of the vPST protein embeddings over each genome. **B–D**) Statistics of genome clusters detected by the Leiden algorithm on a *k*-nearest neighbor graph of the genome embeddings from the test dataset (see Methods): **B**) proportion of genomes clustered, **C**) average number of genomes per cluster, and **D**) total number of clusters. A cluster is only counted if there are at least 2 genomes. **E**) *Top*: Pairwise amino acid identity (AAI) was computed for all pairs of viruses in a cluster and averaged for the entire cluster. Then, the AAI for each cluster was averaged for each method, weighting the clusters by their size. *Bottom*: The data in the top row were scaled by the proportion of genomes clustered from the test dataset. All analyses were performed with the vPST test dataset.

To evaluate if the vPST learned biologically meaningful representations of viral genomes, we compared the genome embeddings produced by the vPST against other protein- and nucleotide-based methods with a quantitative clustering assessment on the vPST test dataset. For protein-based methods, we performed an ablation study comparing unweighted averages of the input ESM2 embeddings and of the vPST protein embeddings over each genome (“ctx-avg” methods). For nucleotide-based methods, we used 4-mer nucleotide frequency vectors, GenSLM^28^ embeddings, and HyenaDNA^29^ embeddings. The latter methods were chosen for both their availability relative to the course of this study and their relevance to genome language modeling, as there have been several recently described nucleotide-based models^30,31^. Notably, both GenSLM and HyenaDNA have also been referred to as genome language models, so we explicitly refer to these as nucleotide language models to distinguish them from our protein-based genome language model. GenSLM was trained to focus on codon-level words in a genome sentence. Thus, to produce GenSLM genome embeddings, we embedded each nucleotide open reading frame (ORF) and then averaged these over each genome. Meanwhile, HyenaDNA is a long-context nucleotide language model that can contextualize up to 1Mb fragments, which is well above the size of most viral genomes. Protein-based methods and HyenaDNA appeared to better reflect the evolutionary relationships among viruses in both the vPST training and test datasets in a qualitative analysis of the genome embeddings in which there are visually distinct clusters of the 4 viral taxonomic realms (Fig. 2A).

To quantitatively evaluate each genome representation, a similarity-weighted *k*-nearest neighbor (*k*NN) graph was constructed from each of the genome embeddings from the vPST test dataset and then clustered using the Leiden algorithm^32^. We considered a range of values for *k*, the number of genome neighbors, and for the clustering resolution, which sets a threshold for how distant connections can be, to better understand the clustering trends with each genome representation (Fig. 2B–D). As expected, increasing *k* from 2 to 50 leads to a greater proportion of viruses clustered with at least 1 other genome (Fig. 2B), increases the average cluster size (Fig. 2C), and decreases the total number of clusters (Fig. 2D). Likewise, increasing the clustering resolution in the Leiden algorithm has the opposite effect when *k* is constant, since more distant connections are pruned in the *k*NN graph (Fig. 2B–D, right column).

We then computed average amino acid identity (AAI) between pairs of genomes in each genome cluster and aggregated the AAI over all genome clusters to assess the quality of the genome clusters. As expected, protein-based methods lead to genome clusters that have higher intra-cluster AAI than nucleotide-based methods (Fig. 2E), suggesting that these methods use overall protein similarity to understand viral genome relationships. Notably, pst-small genome clusters have the highest AAI among all methods (Fig. 2E). However, when penalizing high rates of genome singletons, pst-large genome clusters have the highest AAI (Fig. 2E). Importantly, this implies that pst-large not only clusters viral genomes based on protein similarity but also can relate the largest proportion of genomes. Additionally, most methods also outperform the baseline of clustering viruses specifically using AAI at the genus or family level (Fig. 2E, “AAI-” lines). Further, evaluating the taxonomic purity of both the viruses and their hosts across the genome clusters does not strongly separate any method (Extended Data Fig. 4). This may suggest that current viral taxonomy is not as informative for understanding viral-viral relationships across diverse sets of viruses compared to AAI, which is based on more intrinsic information to the viral genomes. Further, the proportion of viruses with a predicted host is low (Extended Data Fig. 2D), which may also skew this analysis.

### The vPST detects important viral protein functions, including identifying new potential hallmark proteins

**Figure 3.**
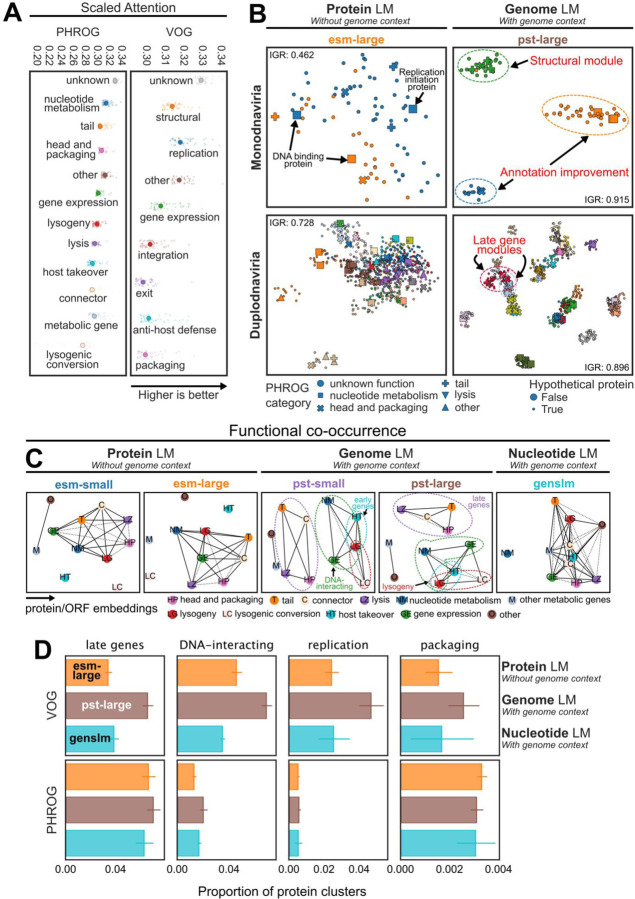
The vPST leverages genomic context to learn protein function relationships. **A**) Scaled attention from pst-large normalized to compare across genomes with differing numbers of proteins (see Methods) with respect to protein function. Scaled attention is the max scaled attention of all proteins in each of the top 50 sequence identity-based protein clusters (mmseqs2). **B**) UMAP dimensionality reduction plots for 2 genome clusters that were primarily (≥85% of genomes) composed of Monodnaviria (top, 13 genomes, 80 proteins) or Duplodnaviria (bottom, 4 genomes, 682 proteins). Colors indicate protein cluster membership defined by clustering the *k*-nearest neighbor graph of the indicated protein embedding with the Leiden algorithm. Here, pst-large refers to the vPST protein embeddings. “IGR” refers to the average weighted information gain ratio for all protein clusters within each of the two genome clusters as a measure of protein cluster functional purity (see Methods). Shapes indicate the PHROG functional category. **C**) Summary of functional co-clustering based on PHROG annotations. Each connected component was clustered in a co-occurrence graph using the Leiden algorithm with resolution of 1.0. The edges indicate pairs of functional categories that were more enriched in protein clusters defined by clustering the *k*-nearest neighbor graph of the corresponding protein/ORF embeddings (columns) relative to the background distribution of annotation profiles. The length of the edges reflects the degree of enrichment since the networks were visualized using a spring force algorithm. Dotted lines indicate connections that were less enriched than or equal to expected, while solid lines were more enriched than expected. **D**) The proportion of protein clusters that correspond to one of the indicated function modules (columns) when using either the VOG (top) or PHROG (bottom) annotation databases. For B and C, genomes were clustered using pst-large genome embeddings (k=15, clustering resolution=“high”). Proteins were clustered within each genome cluster with k=15 and clustering resolution=“med”. All analyses were generated using the vPST test dataset.

The vPST genome representations are produced as a function of the input protein embeddings that get contextualized by the intermediate PST encoder. Thus, we expected that the biologically meaningful genome embeddings of the vPST should be generated from meaningful protein representations. We first analyzed the attention scores of each protein per genome from pst-large, which are used as importance scores when pooling the vPST protein embeddings for the final genome representation. We considered that the general function of each protein was likely associated with high attention. Indeed, structural proteins (head, packaging, tail) and replication or nucleotide metabolism proteins were generally most attended to by the model (Fig. 3A). This is intuitive since these proteins are essential to viruses and likely reflects their relatively greater abundance in the dataset (Extended Data Fig. 5B). Further, we found a subtle association between the attention scores with the number of proteins belonging to the same sequence identity-based cluster (Extended Data Fig. 5A). This reflects the model assigning a higher weight to proteins seen more frequently.

To quantitatively assess the ability of the vPST to understand protein relationships, we conducted a similar analysis as with the genome clusters. The embedding-based protein clusters were generated using the Leiden algorithm on a similarity-weighted *k*NN graph. To reduce potential noise when clustering, we restricted the set of nearest neighbors to only include proteins from genomes in the same genome cluster, specifically using the hyperparameters that maximized intra-genome-cluster AAI (k=15, resolution=“high”, Fig. 2E). We performed a similar purity analysis of the protein clusters with respect to VOG and PHROG functional categories that did not strongly indicate which protein or genome clustering methods produced the most functionally pure genome clusters (Extended Data Fig. 6). However, clustering the genomes with the ctx-avg-large embeddings tended to perform best for protein cluster functional purity (Extended Data Fig. 6B). This result makes sense because the vPST protein embeddings used for the ctx-avg-large genome embeddings are the last time the vPST directly considers protein information. Additionally, vPST protein embeddings led to the overall highest protein functional purity.

To identify cases where the vPST outperforms the input ESM2, we visualized the protein embeddings from 2 representative genome clusters primarily (≥85% of genomes) composed of Monodnaviria or Duplodnaviria using the large embeddings (Fig. 3B). In the Monodnaviria cluster, there are DNA binding proteins that esm-large did not cluster together, reflecting the underlying sequence divergence of these two proteins (35.5% sequence identity over ~71% coverage). However, pst-large clustered these proteins with a replication initiation protein, suggesting a detection of broad functional relationships. Furthermore, the esm-large embeddings clustered various structural proteins together with these DNA-interacting proteins that pst-large notably clustered into distinct clusters. There are additionally numerous proteins unable to be annotated by PHROG (Fig. 3B) or VOG (Extended Data Fig. 5C) that cluster with annotatable proteins regardless of protein embedding used. Similar visual patterns exist for the Duplodnaviria cluster, which prompted us to consider if these were general phenomena of the vPST protein clustering.

### The vPST co-clusters related protein functions into function modules

Given that the vPST leverages genomic context, we suspected that the vPST would be equipped to identify groups of associated protein functions that reflect the underlying genome organization. For example, the late genes encoding for structural, packaging, and lysis proteins are adjacent and transcribed by a single promoter in the Lambda genome^33^. We, therefore, assessed protein function co-clustering patterns. For each protein cluster, we calculated the number of times pairs of proteins belonging to different PHROG functional categories co-clustered against the number of times each pair of categories would be expected to co-cluster based on the underlying distribution of the PHROG database categories. The resulting enrichment networks showed that both vPST models could group proteins based on broader function modules (Fig. 3C), regardless of the genome embedding used for genome clustering (Extended Data Fig. 7). For example, tail, head and packaging, connector, and lysis proteins, which are notably late gene proteins, consistently co-clustered above background in vPST protein clusters. Additionally, DNA-interacting (nucleotide metabolism, lysogeny, and gene expression), early gene (host takeover, lysogeny), and lysogeny (lysogeny, lysogenic conversion) function modules were enriched in vPST protein clusters. Interestingly, regardless of how the genomes were clustered, using ESM2 protein embeddings to cluster the proteins did not lead to interpretable functional modules emerging (Fig. 3C, Extended Data Fig. 7). Additionally, while some functional relationships were detected in the GenSLM ORF clusters, this was not consistent depending on how the genomes were clustered. These results were also consistent with the proportion of protein clusters that we considered as belonging to these function modules such as late genes, DNA-interacting, replication, and packaging (Fig. 3D). Notably, protein clusters that belonged to these function modules made up a greater proportion of vPST protein clusters than ESM2 or GenSLM clusters when using VOG annotations, regardless of how the genomes were clustered (Extended Data Fig. 8A). The effect is less pronounced with PHROG annotations (Extended Data Fig. 8B), but this difference may be attributed to the overall decrease in functional annotation with the PHROG database (Extended Data Fig. 5B), which led to excluding a greater number of protein clusters that belonged to each functional module. These data demonstrate that considering genome context better enables the vPST to detect broader functional associations implicitly encoded in viral genome organization.

### The vPST expands our understanding of proteins of unknown function

**Figure 4.**
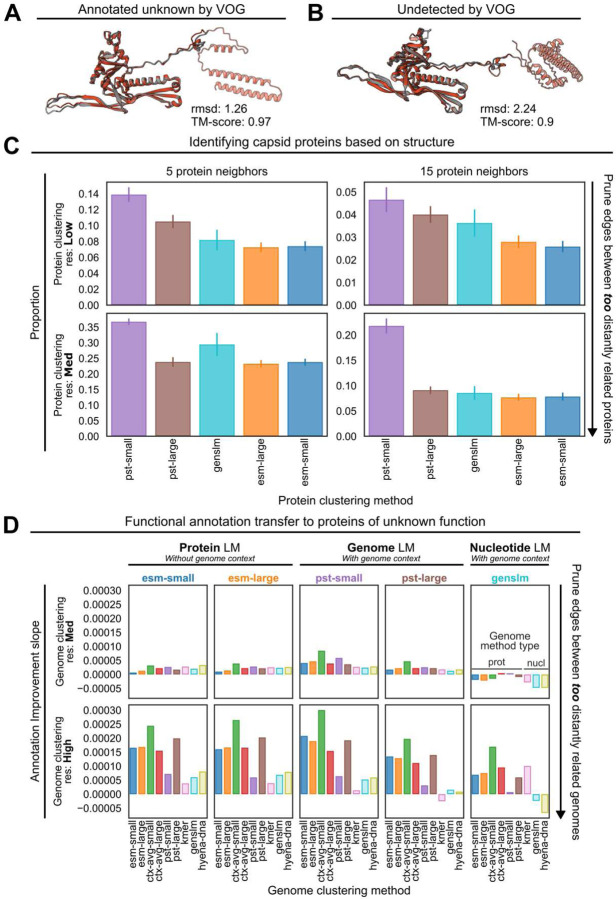
The vPST expands functional annotation of hypothetical proteins. **AB**) Structural alignments with the HK97 major capsid protein (PDB: 2FS3, gray) for a protein annotated by VOG as unknown (**A**, “IMGVR_UViG_2851668853_000002|2851668853|2851668853|1181413-1220308_35”) and another undetected by VOG (**B**, “IMGVR_UViG_3300036770_002539|3300036770|Ga0310126_0001736_19”). The red cartoon diagrams are the query proteins from our dataset and were chosen due to being the most similar to the HK97 capsid protein from each category. **C**) The average proportion of proteins unannotated by VOG clustering with annotated capsid proteins that have structural homology with known capsid folds. Structural homology was detected using foldseek searching against the Protein Data Bank database. Error bars represent the standard deviation over the embedding used for genome clustering. Values are only comparable within each subpanel. **D**) Sensitivity of annotation transfer from annotated to nearby unannotated proteins over the choice of *k* nearest neighbors for genome clustering. Instances of annotation transfer were detected if the nearest protein (based on cosine distance of the protein/ORF embeddings) to each unannotated protein had a VOG annotation. All analyses performed with genomes and proteins from the test dataset.

Interestingly, hypothetical proteins unable to be annotated by either the VOG or PHROG databases were considered the most important by pst-large (Fig. 3A). One explanation is that since proteins of unknown function make up 70–90% of all proteins in the vPST test dataset (Extended Data Fig. 5B, Supplementary Table 2), it is likely that there are true viral hallmark structural and replication proteins that have diverged at the sequence level among the unannotated proteins. To understand if the vPST uses more than sequence-level information to relate proteins, we investigated whether unannotated proteins that cluster with detectable capsid proteins contained conserved capsid-like structural folds as evidence that these unannotated proteins are indeed capsid proteins. We filtered the proteins from the test viruses to maintain proteins belonging to protein clusters that contained only annotated capsid proteins or hypothetical proteins. We then used foldseek^34^ and ProstT5^35^ to translate this protein set into a structural alphabet for searching against Protein Data Bank^36^ structures for structural homology. To validate the structural reasoning of this approach that does not directly infer a protein structure, we independently aligned the structures of the reference HK97 major capsid protein^37^ with two different AlphaFold 3-predicted^38^ structures using the most structurally similar proteins from our dataset: one that was detected by a VOG profile with unknown function (Fig. 4A, pTM=0.66) and one undetected entirely (Fig. 4B, pTM=0.6). The strong alignments indicate that our workflow can accurately identify capsid-fold-containing proteins from the protein sequence alone. Using this approach, the vPST models generally showed the greatest average proportion of unannotated proteins with structural homology to known capsid proteins (Fig. 4C), regardless of how the proteins or genomes were clustered (Extended Data Fig. 9A). GenSLM ORF embeddings were also better than the ESM2 protein embeddings for this task, likely due to being pretrained on microbial genomes, which would contain some viral sequences, and finetuned on SARS-CoV-2 genomes.

We next considered that similarity in embedding space could be used to propagate functional labels from annotated to unannotated proteins. To evaluate the annotation transfer ability of vPST, we first performed a nearest neighbor sensitivity analysis. For all unannotated proteins in the test set, we identified the nearest protein within each genome cluster using cosine distance of each protein embedding. If the nearest protein was annotated by VOG, we scored that as an improvement in annotation. Protein-based embeddings outperformed the GenSLM ORF embeddings for transferring annotations, regardless of how the genomes were clustered (Extended Data Fig. 9B). Additionally, the rate at which this annotation improvement increases as more genome neighbors are considered showed that the vPST was more sensitive (Fig. 4D). Specifically, clustering genomes with ctx-avg-small or pst-large genome embeddings led to the greatest rate of improvement as more genome neighbors are allowed. Interestingly, when nucleotide methods were used for the genome clustering or the protein distance searches, the rate decreased, suggesting that adding more genome neighbors impedes annotation transfer. This may be due to the limited range of nucleotide information in capturing distant relationships. This means that as the nucleotide-based genome clusters increase in size, the nearest neighbor in ORF embedding space for an unannotated protein is just another unannotated protein. Further, considering only the single nearest protein is a conservative baseline. It would be possible to improve these results not only by considering more protein neighbors but also by finetuning the vPST with a protein annotation task.

### The vPST can be applied toward viral host prediction

**Figure 5.**
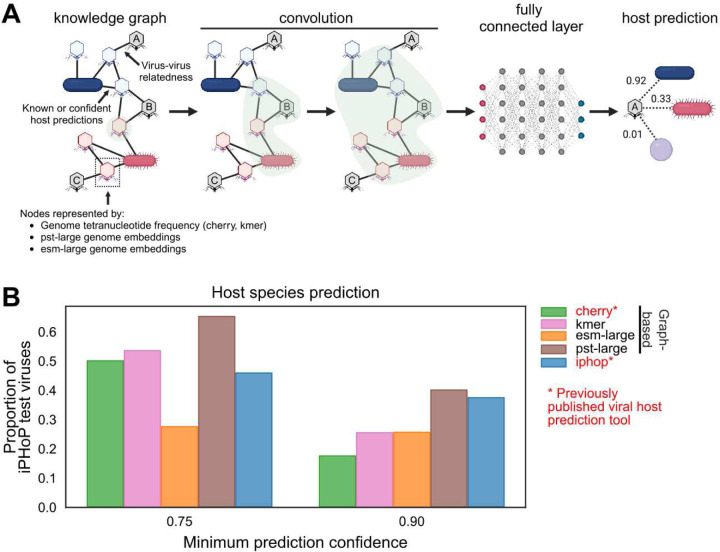
The vPST improves host prediction. **A**) Graph neural network approach for host prediction developed by CHERRY. The node representations are swapped out to the corresponding data type. **B**) Proportion of iPHoP test viruses whose true host species is predicted above the indicated confidence threshold. None of the 1636 iPHoP test viruses were filtered for similarity to those in the vPST training set. The graph-based models were trained in this study, while “iphop” represents the results of iPHoP on the test set.

Since we expect that the vPST can be used as a general-purpose model for downstream viromics tasks, we used the pst-large genome embeddings for viral host prediction as a proof-of-concept (Extended Data Fig. 10A). We adopted and modified a graph framework described previously^39^ that models this scenario as a link prediction task in a virus-host interaction network. Briefly, the objective is to predict for any pair of virus and host whether there should be a link, indicating a prediction for infection of that host by the corresponding virus (Fig. 5A). This task can be performed by a Graph Neural Network (GNN), which uses a form of convolutions to aggregate local (more related) parts of the graph to improve link prediction.

We implemented a variant of the GNN-based CHERRY algorithm^39^ (Fig. 5A), swapping out the node (genome) embeddings of both viruses and hosts with either ESM2, vPST, or the tetranucleotide frequency (kmer) vectors that CHERRY uses. Although this design is likely suboptimal for vPST, which has embeddings specialized for viruses but not hosts, it enables a direct comparison of the choice of genome embedding instead of various virus-host genome embedding combinations. We then trained these models using the training dataset of the host prediction tool iPHoP^40^ to compare with previously published work (Extended Data Fig. 10A). Then, each trained model and iPHoP were evaluated using the same iPHoP test dataset. We evaluated whether the true host species for each test virus could be identified with high confidence (Fig. 5B). The model using vPST genome embeddings outperformed all other methods at the host species level, although the margin between vPST and iPHoP was small when retaining predictions ≥ 0.9 confidence. Although there are viruses in the iPHoP test set that are similar to those in the vPST training set (Extended Data Fig. 10B), excluding these viruses does not change the overall results (Extended Data Fig. 10D). Additionally, when evaluated at broader host taxonomic levels, the kmer model performed the best, with CHERRY and vPST close behind (Extended Data Fig. 10D). The kmer model notably includes implementation-specific changes to CHERRY that appear to enable greater performance. Further, the lower vPST performance at broader host taxonomic levels could be explained by the fact that the vPST genome embeddings were not tuned for hosts. However, the ESM2-based model, which is more comparable to vPST, does not perform well when evaluated at any confidence threshold or host taxon rank. This directly demonstrates the importance of training on viral datasets for viromics tasks.

## Discussion

Here, we presented the PST framework for modeling genomes as sets of proteins, where each protein is initially represented by information-rich ESM2 protein embeddings. The PST contextualizes the input protein embeddings and subsequently yields genome representations as weighted averages of contextualized protein embeddings, which can be targeted toward either protein-level or genome-level downstream tasks. When pretrained on a large, diverse dataset of viral genomes, the vPST demonstrated superior ability in understanding relationships among viral genomes (Fig. 2E). At the protein level, the vPST protein embeddings demonstrated patterns of broad function grouping, consistently clustering late gene proteins together (Fig. 3B). Additionally, vPST often clustered capsid-fold-containing proteins that could not be annotated by VOG with annotated capsid proteins (Fig. 4A), suggesting that vPST uses inferred structural information for relating proteins. The vPST further showed high sensitivity for annotation transfer (Fig. 4B). Performance for these protein-level tasks could be further improved by finetuning the ESM2 pLM with viral sequences and by training a vPST with a dual objective that more directly considers protein-protein and genome-genome relationships. Finally, when applied toward a viral host prediction task, the vPST genome embeddings were able to detect the true host species for the greatest number of viruses when compared against two previously published host prediction tools (Fig. 5B). We notably refrained from overanalyzing the subtle differences in performance in the proof-of-concept host prediction task since there are numerous training techniques beyond the scope of our work that could have resulted in a superior vPST-based host prediction model. It is, therefore, important to emphasize that the vPST-based host prediction model performed on par with (and sometimes better than) existing host prediction tools without the vPST being initially tasked with host prediction and without significant training time.

**Figure 6.**
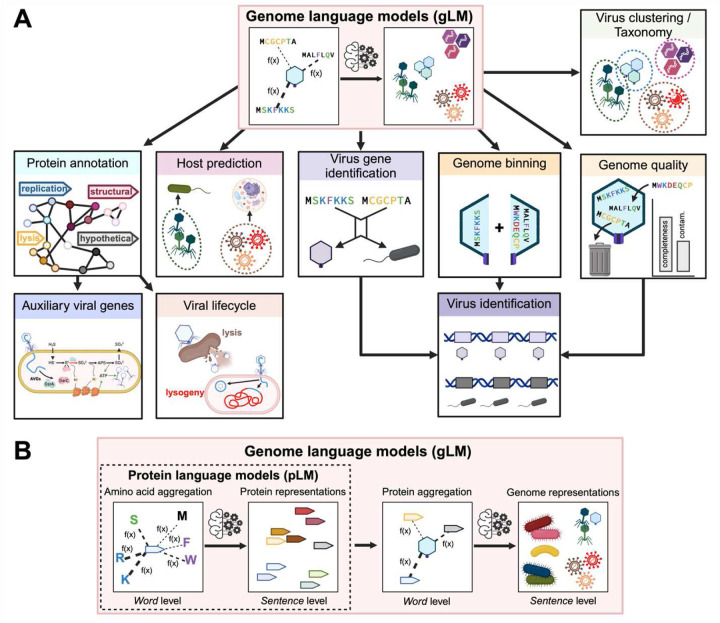
The PST can be a general-purpose microbial and viral genome language model. **A**) Potential downstream tasks of the pretrained vPST that represent commonly desired steps of a typical computational viromics pipeline. **B**) Example workflow of a genome language model based on the PST that could incorporate both microbial and viral input genome datasets.

It is imperative to reiterate that this superior performance in a variety of viromics tasks emerged despite not training the vPST with these objectives. Taken together, our results indicate that the vPST is suitable as a foundation model for common viromics tasks, such as virus identification, taxonomy, host prediction, protein annotation, genome binning, etc. (Fig. 6A). We anticipate that more thorough studies for downstream viromics problems will benefit from starting from our pretrained vPST. Additionally, finetuning the vPST can bring even greater performance for these downstream tasks. For example, finetuning an end-to-end host prediction model with a virus-host dataset would likely significantly improve predictive power compared to what we observed. Further, the iPHoP training dataset has limited diversity (Extended Data Fig. 10C), which could suggest that the results here are not representative of true performance. Nonetheless, our work has provided a guideline for a standalone vPST-based host prediction tool.

There has been growing caution around biological foundation models due to potential biosecurity threats such as generating novel pathogenic viruses or guiding gain-of-function viral mutations. For example, the AlphaFold 3 web server does not allow predictions for certain viral proteins^38^, Evo excluded viruses with eukaryotic hosts from its pretraining data^41^, and ESM3-open filtered viral sequences and select agents from its training sets^42^. While developing vPST, we have assessed the ethical implications of this viral foundation model and are having independent experts consider these impacts before releasing the vPST code and model weights. We, however, perceive the vPST to have a low biosecurity risk. First, only 0.2% of the vPST training viruses infect humans. Of these, only 4 are on the CDC’s list of bioterrorism agents (https://emergency.cdc.gov/agent/agentlist-category.asp; Filoviridae viruses: Ebolavirus and Marburgvirus), and 10 more are under surveillance by the National Respiratory and Enteric Virus Surveillance System (https://www.cdc.gov/nrevss/php/dashboard). Further, only 1% of the training viruses infect mammals, which would be the most likely viral reservoirs that could spillover into human populations. Since our model was not trained considering host identity, the low abundance of these viruses in the training dataset likely minimizes their influence on the learned vPST embeddings. Second, the lowest resolution of the vPST is at the protein level, meaning that it would be difficult to reverse engineer a *de novo* viral genome using the vPST. While a nucleotide language model reported the ability to generate *de novo* bacterial virus genomes^31^, the similarity of these genomes to the training dataset was not investigated. One pitfall is that the model could have been generating trivially *de novo* genomes that do not differ substantially from the training data. Reverse engineering genomes from our protein-based work is further complicated by the complexities of how human viruses tend to encode and express genes (overlaps, alternative starts, alternative splicing, post-translational processing, etc.). These molecular biology issues likely mean that achieving *in vivo* activity of a generated viral genome would be challenging. We, therefore, perceive that the demonstrated and potential future benefits (Fig. 6A) of our work to advance our understanding of viruses outweigh any hypothetical threats that would require significant resources to unleash.

Finally, our PST architecture, while trained on viral proteins and genomes for this study, is agnostic to the source of the proteins and type of genomes. The only requirements of our framework are the ordered protein sequences and genome strand of each ORF. These requirements are more easily satisfied by microbial genomes, where computational ORF calling is both accurate and common. However, our PST could theoretically work with large enough datasets of experimentally determined ORFs from eukaryotes as well. Nonetheless, we propose that our PST implementation is equally appropriate for developing a microbial foundation model to solve challenges in microbial genomics (Fig. 6B), which notably also include poor protein annotation rates and high sequence divergence. In fact, our foundation vPST model was still useful for host genome representations in the virus-host prediction task (Fig. 5), despite only being trained on viruses.

## Online Methods

### Viral genome datasets

We acquired viral genomes from 12 different publicly available sources^1,17–27^ as a training dataset. For GTDB (r202), we used PhageBoost^44^ (v0.1.7) with default settings to identify integrated proviruses, filtering predictions that did not encode at least 20 proteins. We then filtered genomes that were not considered complete or high-quality as defined by CheckV^45^ (v1.0.1). We then dereplicated this set of genomes using a custom workflow. We first used skani^46^ (v0.1.0 sketch: --fast) to compute pairwise average nucleotide identity (ANI) between all pairs of viruses. We constructed a graph where edges connected viruses with ≥95% ANI and ≥50% genome coverage of the alignment for both genomes. The edge weights were the product of ANI and coverage. We then used the Markov clustering algorithm^47^ (mcl v14-137 -I 2.0) to cluster this graph, taking one genome from each cluster at random as a representative genome. For the test dataset, we chose the most complete, least contaminated, and longest genome for each viral operational taxonomic unit in IMG/VR v4^11^, ensuring that each representative was considered high-quality by CheckV. We then dereplicated this putative test dataset with the training dataset using a similar approach as above with skani (--slow, ≥95% ANI, ≥85% coverage) and mcl. We kept all viruses that did not cluster with training viruses. For both datasets, we filtered out viruses predicted to encode only 1 protein. The final number of viral genomes was 103,589 for the training dataset and 151,255 for the test dataset.

For all viruses, we predicted protein open reading frames (ORFs) using the Python bindings of prodigal called pyrodigal^48^ (v2.3.0) for single-contig viruses and prodigal-gv (v2.11.0) for viral metagenome-assembled genomes (vMAGs). We did not consider the updates made by prodigal-gv^49^ (include gene models for giant viruses and viruses using alternative genetic codes) to be substantial enough to apply to the entire dataset given the scale and distribution of the data. This led to 6,391,562 proteins for the training dataset and 7,182,220 for the test dataset.

For the training viruses, viral taxonomy not provided by IMG/VR v3 was assigned using geNomad^49^ (v1.5.0) to get labels that were consistent with the current standards. For the test viruses, we used the provided taxonomic labels since they were consistent with current standards, and most were predicted using geNomad also. We did not perform host prediction on these viruses, so host labels were either predicted by the source database or are known due to integrated provirus prediction. The summary of information for the training and test viruses can be found in Supplementary Table 1.

### ESM2 protein language model embeddings

PyTorch (v2.1.0)^50^ and fair-esm^3^ (v2.0.0) were used to obtain protein embeddings. We refer to the ESM2 models “esm2_t6_8M_UR50D” (6 layers, 8M parameters, 320-dimensional embedding) and “esm2_t30_150M_UR50D” (30 layers, 150M parameters, 640-dimensional embedding) as “esm-small” and “esm-large”, respectively. The amino acid embeddings in each protein were averaged for a single 𝑑-dimensional vector. For proteins longer than 20,000 amino acids, the sequence was split in half, and the embeddings for each half were then averaged for the final embedding. This only affected 1 bacterial protein in the host prediction analysis.

### The Protein Set Transformer model architecture

The Protein Set Transformer (PST) was built using PyTorch (v2.0.0), PyTorch Geometric^51^ (v2.3.1), and PyTorch-Lightning (v2.0.7). The PST draws inspiration from deep learning of set-structured data like the SetTransformer^12^ while using modifications that are specific to pointsets^13^, which are sets whose items are 𝑑-dimensional vectors. The PST, thus, models genomes as a set of proteins $G_{i}$ where $g_{ij}\in G_{i}$ is the 𝑗th protein in the 𝑖th genome. Each protein is initially represented by its 𝑑-dimensional ESM2 protein embedding, with $G_{i}\in R^{n_{i}\times d}$ where $n_{i}$ is the number of proteins encoded in genome $G_{i}$. We did not finetune the ESM2 models, so the ESM2 embeddings were used as frozen inputs. For each protein $g_{ij}$, learnable embeddings for both the position in the genome and for the encoding strand were concatenated to the ESM2 embeddings. The positional embeddings for proteins were relative to the positions of the proteins in each genome and are used so the model learns relative ordering of proteins. For fragmented genomes such as viral metagenome-assembled genomes (vMAGs), the scaffolds were randomly oriented such that all proteins were numbered continuously from the randomly chosen starting scaffold.

To account for the large variation in the number of proteins encoded by each genome, we used a memory-efficient graph-based implementation that considers each genome as a graph and each protein as nodes in the genome graph. Notably, each individual genome matrix $G_{i}$ is stacked for each minibatch, so there is no padding. Then, an indexing pointer keeps track of the offsets (number of rows/proteins) for each genome for efficient access. For memory efficiency and to model real fragmented genomic data, we break each genome graph into subgraphs whose node sets include 15–50 mutually exclusive, contiguously located proteins. The size of each subgraph was tuned and is, thus, fixed. These nodes are all fully connected in each subgraph such that all proteins in each genome subgraph attend to each other in the PST encoder. We prevent subgraphs with 1 node by adding possible singleton node cases to the previous subgraph. The subgraph size (“chunk size”) hyperparameter is constant for all genomes. Thus, a minibatch of 𝑁 genomes is represented by a single graph $𝓖=\left(X,E\right)=Stack\left(G_{i}\right)$ where X is the total number of proteins encoded by the N genomes. E is the total number of protein-protein edges and is a function of the subgraph size and number of proteins per genome.

The PST uses the encoder-decoder paradigm previously described with the SetTransformer^12^. The encoder uses multi-head self-attention to contextualize each protein by the other proteins within the same genome. Then, the decoder uses multi-head attention pooling to summarize the genome as a weighted average of contextualized protein embeddings. To contextualize the proteins in each genome, we used a graph-based implementation of multi-head scaled-dot product self-attention^14^ in each layer of the PST encoder:

$$\alpha_{ij}=\text{GraphSoftmax}\left(\frac{\left(W^{Q}x_{i}\right)\left(W^{K}x_{j}\right)}{\sqrt{d}}\right)$$

$$\text{MultiHeadAttn}\left(𝓖\right):x_{i}^{\left(l+1\right)}=W^{Q,\left(l\right)}x_{i}^{\left(l\right)}+\sum_{j\in 𝓝\left(i\right)\cup \left\{i\right\}}\alpha_{ij}^{\left(l\right)}W^{V,\left(l\right)}x_{j}^{\left(l\right)}$$

where $x_{i}^{\left(l\right)}$ is the embedding vector for the 𝑖th protein at the lth encoder layer, d is the protein embedding dimension. Likewise, $W^{\cdot ,\left(l\right)}$ is the weight matrix for the query, key, and value at the lth encoder layer. $𝓝\left(i\right)$ is the set of protein neighbors for the ith protein in the same genome subgraph. $A_{ij}$ is the scaled-dot product attention calculation. GraphSoftmax is a modified softmax function that only normalizes the attention values within the set of subgraphs that belong to the same genome. Thus, only proteins in the same subgraph attend to each other, but the attention values are normalized by all proteins in the genome. To enable multi-head attention, we split the input protein embeddings in the same number of chunks as the number of attention heads along the embedding dimension. After the self-attention calculation, we concatenate the outputs from each head back together. Further, we followed a pre-normalization strategy in which we normalized the input protein embeddings before the linear layers. Specifically, we used GraphNorm (implemented in PyTorch-Geometric) normalization operator that normalizes the proteins embeddings only within each genome. Additionally, we used the corresponding skip connections in which untransformed inputs are added to values post-attention. A full PST encoder layer can thus be mathematically represented as the following set of equations (1):

(1) $$X^{1}=\text{GraphNorm}\left(X^{0}\right)\;X^{2}=\text{MultiHeadAttn}\left(𝓖\right)\;X^{3}=X^{0}+X^{2}\;X^{4}=\text{GraphNorm}\left(X^{3}\right)\;X^{5}=\text{FF}\left(X^{4}\right)\;X^{6}=X^{3}+X^{5}$$

where X represents the intermediate protein representations and $X^{0}$ is the input protein embeddings in the stacked batch. FF represents a 2-layer feedforward network with GELU (Gaussian error linear unit^52^) activation and dropout after each layer. After the full PST encoder, a final GraphNorm operation was applied.

The PST decoder uses multi-head attention to compute a per-protein attention score to be used as the weights for a weighted average of protein embeddings over each genome. As described previously^12^, multi-head attention pooling uses a learnable 𝑑-dimensional seed vector S as the query when computing attention. During the attention calculation, the contextualized protein embeddings $X^{C}$ output from the PST encoder are projected onto S:

$$Attn\left(X^{C},S\right)=\text{GraphSoftmax}\left(\frac{\left(W^{Q}S\right)\left(W^{K}X^{C}\right)}{\sqrt{d}}\right)\times \left(W^{V}X^{C}\right)$$

The attention values from this projection are used to weight $X^{C}$. After re-weighting, $X^{C}$ is averaged over each genome to produce the final genome outputs. The full set of PST decoder equations is similar to the encoder (Equation 1):

$$X^{0}=\text{GELU}\left(WX^{C}\right)\;X^{1}=\text{GraphNorm}\left(X^{0}\right)\;X^{2}=\text{Attn}\left(X^{1},S\right)\;X^{3}=X^{0}+X^{2}\;X^{4}=\text{GraphNorm}\left(X^{3}\right)\;X^{5}=\text{FF}\left(X^{4}\right)\;X^{6}=X^{3}+X^{5}\;X^{7}=\text{GraphPool}\left(X^{6}\right)\;X^{G}=\text{FF}\left(X^{7}\right)$$

where W is the weights of a linear layer. GraphPool is a pooling (mean) operator over each genome graph that averages the contextualized weighted protein embeddings for each genome. Each FF is a different 2-layer feedforward network with GELU activation and dropout after each layer. $X^{G}$ is the final genome embeddings. See Extended Data Fig. 1 for a pictorial representation the PST architecture.

### Training the viral Protein Set Transformer foundation model with triplet loss

The foundation viral Protein Set Transformer (vPST) model was trained using a self-supervised triplet loss objective $𝓛\left(𝓖\right)$ as described previously^13^:

$$D\left(G^{a},G^{p},G^{n}\right)={\left[{\left\|f\left(G^{a}\right)-f\left(G^{p}\right)\right\|}_{2}^{2}-\omega_{i}{\left\|f\left(G^{a}\right)-f\left(G^{n}\right)\right\|}_{2}^{2}+\alpha \right]}_{+}$$

$$𝓛\left(𝓖\right)=\frac{1}{2N}\sum_{i=1}^{N}C_{i}\left[D\left(G_{i}^{a},G_{i}^{p},G_{i}^{n}\right)+D\left(G_{i}^{a}+G_{i}^{\prime p},G_{i}^{\prime n}\right)\right]$$

where $G_{i}^{a}$ is the 𝑖th genome treated as an anchor point, $G_{i}^{p}$ is the positive genome for the 𝑖th genome, $G_{i}^{n}$ is the negative genome for the ith genome, and $G_{i}^{\prime }$ is the augmented genome for the ith genome created using the PointSwap sampling method^13^. $f\left(\cdot \right)$ is the function modeled by the full PST neural network, and ${\left\|x-y\right\|}_{2}^{2}$ is the L2 (Euclidean) distance between the vectors x and y. $C_{i}$ is class weight to amplify the contribution to the loss for classes that are less abundant than others. We used the viral realm of each virus as the class and compute $C_{i}$ as an inverse abundance frequency. Suppose that the 𝑖th genome belongs to viral realm k, then the class weight is computed as:

$$F_{k}=\frac{n_{k}}{N}$$

$$C_{i}=\frac{1}{F_{k}}$$

where $n_{k}$ is the number of genomes in the training dataset belonging to viral realm k out of N total genomes.

To account for the self-supervised choice of the negative genome, the scale factor $\omega_{i}$ reweights the anchor-negative distance according to the following exponential decay equation:

(2) $$\omega_{i}=\exp\left(-\frac{\text{CD}\left(G_{i}^{a},G_{i}^{n}\right)}{2{\left(c\sigma \right)}^{2}}\right)$$

where $\text{CD}\left(X,Y\right)$ is the Chamfer distance between the genomes X and Y, c is a scaling factor, and σ is the standard deviation of all Chamfer distances. ${\left[\cdot \right]}_{+}=\max(0,\cdot )$, which means that there is no contribution to the loss function for cases where the positive genome is already closer to the anchor genome than the negative by a margin of α. Thus, α is the farthest distance the negative genome needs to be from the anchor compared to the positive genome. This restraint notably prevents representation collapse that could occur in the naïve case of embedding the anchor and positive genomes in the same position.

For a training minibatch, positive mining occurs in the input ESM2 embedding space using Chamfer distance, before the PST forward pass and before concatenating positional and strand embeddings. The Chamfer distance $\text{CD}\left(X,Y\right)$ between genomes X and Y always uses the input ESM2 embeddings and is defined as follows:

(3) $$\text{CD}\left(X,Y\right)=\frac{1}{\left|X\right|}\sum_{x\in X}\underset{y\in Y}{\min}{\left\|x-y\right\|}_{2}^{2}+\frac{1}{\left|Y\right|}\sum_{y\in Y}\underset{x\in X}{\min}{\left\|x-y\right\|}_{2}^{2}$$

where x∈X are the proteins from genome X and y∈Y are the proteins from genome Y. Intuitively, this means that the positive genome is defined as the most similar genome based on cumulative distance of ESM2 protein embeddings, which should choose a positive genome that encodes the most similar proteins.

Negative mining occurs in the PST embedding space and requires the positive genome for a semi-hard sampling scenario. The only candidates for a negative genome are those that are farther than the positive genome in the PST embedding space using Euclidean distance, and we choose the first genome that is farther than the positive as the negative in the semi-hard case. In cases where there are no genomes farther than the positive genome in the PST embedding space, such as at the beginning of training when the model weights have not been well-optimized, we loosen the semi-hard sampling requirement and choose the genome closest to the positive genome as the negative genome. Since negative mining is self-supervised, we use the exponential decay reweighting factor $\omega_{i}$ to down-weight poor choices of a negative genome that are actually very similar to the anchor genome. Notably, the $\omega_{i}$ reweighting factor depends on the Chamfer distance (Equation 2) and, subsequently, the input ESM2 embeddings. Thus, we implicitly consider the ESM2 embeddings as a ground truth for protein representation when mining both the positive and negative genomes.

### PointSwap sampling

When training the vPST, we used the data augmentation technique PointSwap sampling^13^. During positive mining, we keep track of the most similar protein from the positive genome $X^{p}$ for each protein in the anchor genome x∈X (Equation 3) as the flow $x_{i}\to x_{j}^{p}$. We create the augmented genome $X^{\prime }$ as follows:

$$X^{\prime }=\text{PointSwap}\left(X,X^{p}\right)=\left\{x_{0}^{\prime },...,x_{n_{i}}^{\prime }\right\},\;\text{where}\;x_{i}^{\prime }=\left\{\begin{array}{cc} x_{j}^{p} & \text{if}\;u_{i}<p\\ x_{i} & \text{otherwise} \end{array}\right.$$

where $u_{i}$ is a set of samples from a standard uniform distribution [0,1] and 𝑝 is a rate of protein swapping between genomes. This means that the augmented genome $X^{\prime }$ differs from the anchor genome by swapping related proteins with the most related positive genome, which intuitively mimics genetic variation. To form an augmented triplet with the augmented genome as the positive genome, the negative genome is selected from the set of augmented genomes in a minibatch using the procedure described above.

### Modified Leave-One-Group-Out cross validation and hyperparameter tuning

To optimize the model hyperparameters (Supplementary Table 3), we used Optuna^53^ (v3.3.0) to iteratively sample hyperparameters in a direction that optimizes the objective function using a Bayesian Tree-structured Parzen Estimator method. Model performance was evaluated using a modified version of the Leave-One-Group-Out (LOGO) cross validation (CV) strategy. Here, we considered the viral taxonomic realm to be the group with 5 total groups: Duplodnaviria, Monodnaviria, Riboviria, Varidnaviria, and Unknown / Other. We modified the LOGO strategy to always include Duplodnaviria in each training fold, since this group of viruses accounted for 65.4% of the training dataset. This resulted in training 4 separate models validated on the remaining viral realms. Each of the 4 folds were synchronized during training to enable overall performance monitoring as the average of each fold. This enabled real-time monitoring of each tuning trial’s performance. Thus, we were able to stop trials early depending on several criteria using the average validation loss of each fold: (1) if the loss plateaued (std of change less than 1e-6) after having trained 3 epochs, (2) if the loss did not decrease by 0.05 within 5 epochs, (3) if the current performance was worse than the median performance of previous trials at the same training epoch, (4) if the model was trained for 20 epochs, (5) if 24 hours passed, (6) of if the loss was not finite. For number 3, this was maintained by the Optuna framework, and we required at least 1 complete trial before this was enabled. In the case of early stopping due to reasons 1, 2, 3, and 6, these trials were marked as pruned and not used by Optuna’s median performance calculation.

In total, we trained 16 complete, 16 failed, and 22 pruned trials using “esm-large” protein embeddings as input and 45 complete, 1 failed, and 29 pruned trials using “esm-small” protein embeddings as input. The only reason for failing was due to out-of-memory errors on A100 80GB vRAM GPUs hosted by the University of Madison-Wisconsin Center for High Throughput Computing^43^. All trials were tuned using 1 GPU since Optuna has limited support for GPU parallelism.

The final performance for each training iteration was the average validation loss from each of the 4 models. Once the triplet loss of the best model setup decreased below 20.0, we chose the best hyperparameter configuration and trained 2 vPST models corresponding to esm-small and esm-large protein embeddings that we refer to as pst-small and pst-large, respectively. Each vPST model was trained on all genomes in the training dataset without validation for 15 (pst-large, 33.7 hours) or 50 epochs (pst-small, 10.2 hours). Training was stopped once the training loss plateaued and did not decrease by 0.05 within 5 epochs. During training of the final models, a learning rate scheduler was used that linearly decreased the learning each epoch, and 50 (pst-large) or 100 (pst-small) minibatches were accumulated before backward passes. We tested batch accumulation sizes of 1, 25, 50, 100, and 250, and the above values led to the best model.

For both tuning and training the final models, gradients were clipped to keep all values below a magnitude of 1.0, and we used mixed precision training, using bfloat-16 data when available. These choices helped stabilize training. Our fold training synchronization strategy and modified LOGO CV approach were implemented in a custom package called “lightning-cv” available from the main model repository. This package heavily relies upon and extends functionality in the lightning-fabric sub-library of PyTorch-Lightning (v2.0.7).

### GenSLM open reading frame (ORF) and genome embeddings

We used the 25M parameter GenSLM^28^ foundation model (“genslm_25M_patric”, downloaded September 2023) for our analyses since the output embedding dimension (512) was on par with other protein and genome embeddings used. The GenSLM foundation model is pretrained only on bacterial and archaeal nucleotide genes where the gene sequences were broken into codons as input. The authors then finetuned the foundation models on a dataset of SARS-CoV-2 genomes. However, it is not clear if only the open reading frames (ORFs) from SARS-CoV-2 were included or if entire viral genomes were used as input during finetuning. This is further complicated by the fact that the protein-coding density of the SARS-CoV-2 genome is 71.2% (based on the NCBI RefSeq reference sequence NC_045512.2). We chose to mimic the pretraining setup and input the protein-coding ORFs for each virus in our datasets. Notably, we used GenSLM as a nucleotide analog of ESM2, producing ORF embeddings akin to the ESM2 protein embeddings. We used these ORF embeddings for protein/ORF analyses and the average of these over each genome as genome embeddings for genome analyses.

### HyenaDNA genome embeddings

We used the HyenaDNA^29^ model with the longest context size (1M nucleotides, “large-1m”, downloaded from HuggingFace in November 2023) that has 6.5M parameters. We converted all non-ACGTN nucleotides to Ns. Genomes larger than 1M nucleotides were split into non-overlapping fragments of 1M nucleotides at most. Then each fragment was tokenized and fed to the “large-1m” HyenaDNA model. The embedding of each genomic fragment was averaged to produce the final genome embedding. We also used this same averaging approach for fragmented genomes (ie vMAGs), where the final genome embedding was the average of each fragment.

### Tetranucleotide frequency vectors as simple genome embeddings

For each genome, we computed tetranucleotide frequency vectors $\left(\left[\begin{array}{ccc} AAAA & \cdots & TTTT \end{array}\right]\right)$ using the bionumpy^54^ package (v1.0.8). We filtered all nucleotides not in the canonical ACGT alphabet before calculation. For RNA viruses, U nucleotides were represented by T for simplicity. For multi-scaffold viruses, these frequency vectors were computed for each scaffold and then averaged over each scaffold. Throughout the paper, these are referred to as “kmer”.

### Clustering genome and protein embeddings

We constructed a similarity-weighted *k*-nearest neighbors (*k*NN) graph. The set of *k*NN was computed using the faiss^55^ (v1.8.0) Python bindings. For genome embeddings, we used the divide-and-conquer IndexIVFFlat search index that splits the input embeddings into $n_{cells}$ Voronoi cells for faster retrieval. For the training dataset (n=103,589 viral genomes), $n_{cells}$ = 2650, and for the test dataset (n=151,255 viral genomes), $n_{cells}$ = 3875. Then, the L2 (Euclidean) distance D was used to identify the closest genome neighbors. The L2 distances were converted to similarity scores 𝑆 using a Gaussian kernel:

(4) $$S=\exp\left(-\frac{D^{2}}{\sqrt{d}}\in \left[0,1\right]\right)$$

where d is the dimensionality of the genome embedding. These similarity scores were used as edge weights in the *k*NN graph.

For protein embeddings, we restricted the *k*NN search to only consider proteins that belong to genomes within the same genome cluster. The protein embeddings were unit normalized such that the L2-norm for each protein embedding equaled 1. Then, we used cosine similarity to select the set of *k*NN, and the cosine similarity scores were used as the *k*NN graph edge weights. For genome clusters with fewer than 78 proteins, cosine similarity was brute-force computed for all pairs of proteins. For larger genome clusters, the IndexIVFFlat partitioning method was used where $n_{cells}=\left\lfloor \frac{n_{proteins}}{39}\right\rfloor$ since 39 is the minimum number of data points per Voronoi cell. Genome or protein clusters were then detected in the similarity-weighted *k*NN graph using the Leiden^32^ algorithm Python implementation of iGraph (v0.11.3). The resolution values we used were 0.1 (“med”) and 1.0 (“high”) for genome clustering and 0.1 (“low”) and 0.5 (“med”) for protein clustering. We do not include singletons as clusters for downstream analyses.

### Genome and protein clustering evaluation

To compare clusters formed using different input embeddings, we computed the cluster-wise average amino acid identity (AAI) between genomes, viral and host taxonomic purity, and protein function purity for each cluster. Each of these cluster-level metrics was weighted by the size of each cluster, specifically including unlabeled genomes or proteins in the size calculation, and then summarized with a weighted average:

$$C_{\text{summary}}=\sum_{i=1}^{n_{\text{clusters}}}C_{i}w_{i}$$

where

(5) $$w_{i}=\frac{n_{i}}{\sum_{i=1}^{n_{\text{clusters}}}n_{i}}$$

and $C_{i}$ is the cluster-level metric.

Finally, the summary score $C_{\text{summary}}$ was weighted by the proportion of non-singletons for the given dataset P to penalize clustering iterations that did not include all genomes or proteins:

$${C^{\prime }}_{\text{summary}}=C_{\text{summary}}\times P$$

Protein functional purity was computed using curated functional categories from VOG or PHROG. To compute purity of clustering (viral or host taxonomy, protein function), we used the information gain ratio I as a proxy for purity. For taxonomic purity, we considered the case of clustering all genomes into a single cluster as the background. For functional purity, we used the distribution of functional categories from the annotation databases as the background. In either case, unlabeled proteins and genomes were excluded during the entropy computation but included for the cluster size weighting. Then we computed 𝐼 as follows:

$$I=\frac{H_{background}-\sum_{i=1}^{n_{\text{clusters}}}H_{i}w_{i}}{H_{background}}\in \left[-h,1\right]$$

where h is the information gain of the background compared to a uniform distribution and H is the information entropy of each cluster with respect to a set of labels related to viral taxonomy, host taxonomy, or protein function. The cluster size weight $w_{i}$ is computed as previously described (Equation 5). Values of I close to 0 indicate clustering patterns with no improvement above background, while values of I close to 1 suggest maximal purity since there are few clusters with multiple labels. It is possible to interpret I as a purity score since the backgrounds are not pure with respect to the labels. Thus, a maximum I means that there is only a single label for a given cluster. I is further weighted by the proportion of non-singletons:

$$I^{\prime }=I\times P$$

### Average amino acid identity (AAI)

We used mmseqs2^56^ (v13.45111) and polars (v0.20.6) to compute the AAI between pairs of viruses at scale. Given the large number of viruses in this study (>250k), we did not exhaustively compute the AAI between all pairs of viruses (~32.5B). Instead, we used heuristics implemented by mmseqs2 to only consider the AAI between viruses that had any protein similarity detectable when using the mmseqs2 search settings: -s 7.5 -c 0.3 -e 1e-3. For each pair of viral genomes, we only retained the best hits for each protein from each genome. Then, AAI was computed as the mean of protein-protein sequence similarities computed by mmseqs2.

### Average amino acid identity (AAI) genome clustering

During calculation of AAI for a pair of viral genomes, we tracked the proportion of shared proteins relative to the total number of proteins from each genome u and v as $S_{u}$ and $S_{v}$, respectively. To cluster viral genomes using AAI, we constructed an edge-weighted graph with edge weights corresponding to:

$$E_{uv}=\min\left(S_{u},S_{v}\right)\times AAI\times 100\in \left[0,1\right]$$

The edge weights, therefore, penalize cases where only a few proteins relative to the total number of proteins in the genome with fewer proteins are used for the AAI calculation. We then applied the Markov clustering algorithm^47^ (mcl v14.137 -I 2.0 for “med” resolution or 4.0 for “high” resolution), which uses edge-weight-guided probabilistic random walks to cluster the AAI graph.

We only considered two levels of clustering, genus-level and family-level, using thresholds previously described^57^. Genus-level clustering sets the minimum AAI to 0.4 and requires either at least 16 shared proteins or $\min\left(S_{u},S_{v}\right)\geq 0.2$. Family-level clustering sets the minimum AAI to 0.2 and requires either at least 8 shared proteins or $\min\left(S_{u},S_{v}\right)\geq 0.1$.

### Protein functional annotation

We used VOG (r219) and PHROG^58^ (v4) databases for the annotation of viral proteins. For VOG, which supplies profile Hidden Markov models (HMMs), we used pyhmmer (v0.9.0) with a bit score cutoff of 40. For PHROG, we used mmseqs2 (v13.45111) with the recommended search settings (https://phrogs.lmge.uca.fr/READMORE.php). In both cases, we kept the best hit for each protein with the max bit score. For each database, we curated the functional categories of each annotation that we describe below.

For PHROG, which already provides an extensive set of 10 categories (including unknown function), we manually readjusted certain categories. Our manual curation of the PHROG database affected 1,937 out of 38,880 profiles. We renamed the following categories for better intuition of the functional category: “DNA, RNA and nucleotide metabolism” to “nucleotide metabolism”, “integration and excision” to “lysogeny”, and “transcription regulation” to “gene expression”. We then dissolved the “moron, auxiliary metabolic gene and host takeover” category for being too broad and relatively unrelated. These 461 profiles were split into the already existing “other”; the newly created “host takeover”, “lysogenic conversion”, “metabolic gene”; and the renamed “gene expression”, “lysogeny”, and “nucleotide metabolism” categories. Generic annotations like “membrane associated protein” and “ABC transporter” were put in the “other” category. We considered proteins involved in host replication and cell division inhibition, superinfection exclusion, anti-sigma factors, and defense against host antiviral proteins to be “host takeover”. Proteins that encoded toxins or antitoxins/resistance proteins were categorized as “lysogenic conversion.” Proteins directly involved in specific metabolic transformations were put in “metabolic gene,” while accessory or generic proteins like “nicotinamide mononucleotide transporter” were considered as “other”. These changes can be found in Supplementary Table 5.

VOG provides very broad categories: “Xr” for replication, “Xs” for structural, “Xh” for host-benefitting, “Xp” for virus-benefitting, and “Xu” for hypothetical proteins. The “Xh” and “Xp” categories are also ambiguous on what specific function the protein may perform. We, therefore, used text pattern matching on the specific HMM annotation descriptions to subdivide all HMMs into 9 categories: anti-host defense, exit, gene expression, integration, packaging, replication, structural, other, and unknown. Briefly, we separated terminases, portal proteins, and head packaging proteins from other structural proteins into a “packaging” category. Lysis, virion export, and budding HMMs were considered collectively as the “exit” group. “Integration” includes both integrases and excisionases as well as transposases. We considered all nucleotide metabolism and genome replication to be part of “replication”. To account for overlap in text matching, we enforced the following hierarchy: structural > packaging > exit > integration > gene expression > anti-host defense > replication > unknown > “RNA polymerases” > other. The final category for each HMM was, therefore, the highest in the hierarchy. We added RNA polymerases that did not indicate if they were replicative RNA-directed or transcriptive DNA-directed at the bottom to put these specific RNA polymerases in the “gene expression” category. Additionally, HMMs without matches were thus considered in the “other” category. The category for each VOG r219 HMM can be found in Supplementary Table 6, and the regex patterns used to categorize each HMM can be found in Supplementary Table 7.

### Protein attention scaling and analysis

We computed the attention values as follows: Let $A_{i,j}\in A_{i}$ be the mean attention score across all attention heads for the jth protein from the ith genome:

$$A_{ij}=\frac{1}{n_{\text{heads}}}\sum_{k=1}^{n_{\text{heads}}}A_{ijk}$$

The sum of per-protein attention values for each genome is 1.0:

$$\sum_{A_{ij}\in A_{i}}A_{ij}=1.0$$

Given $n_{i}$, the number of proteins in the 𝑖th genome, and $n_{k}$, the number of proteins in the 𝑘th genome, and $n_{i}\neq n_{k}$, it follows that $A_{i}$ and $A_{k}$ are not directly comparable since the number of proteins each genome is not the same. More explicitly stated, consider $n_{i}=2$ and $n_{k}=4$, and $A_{i}=\left[\begin{array}{cc} 0.5 & 0.5 \end{array}\right]$ and $A_{k}=\left[\begin{array}{cccc} 0.5 & 0.3 & 0.05 & 0.15 \end{array}\right]$. For the genome i, the model has randomly split attention to both proteins, while for genome k, the model clearly has attended to the first protein more than the others, despite the numerical values being equivalent.

Therefore, to compare the vPST attention values per protein for each genome, we normalized the attention scores. We considered the background case for the attention distribution $A_{i}$ to be a uniform distribution, ie $A_{i}\sim U\left(0,1;n_{i}\right)$ where $U\left(0,1;n_{i}\right)$ is a standard uniform distribution with probability $\frac{1}{n_{i}}$ of attending any of the $n_{i}$ proteins. We then computed the distance between $A_{i}$ and $U\left(0,1;n_{i}\right)$ using the normalized Kullbach-Leibler (KL) divergence:

$$D_{i}=\frac{H_{i}^{U\left(0,1;n_{i}\right)}-H_{i}^{A_{i}}}{H_{i}^{U\left(0,1;n_{i}\right)}}\in \left[0,1\right]$$

$H^{X}$ is the entropy of the probability distribution X:

$$H\left(X\right)=-\sum_{x\in X}p\left(x\right)\log_{2}p\left(x\right)$$

We then rescale all per-protein attention values in $A_{i}$ by the KL-divergence $D_{i}$ to down-weight misleadingly large attention values that are uniformly (randomly) distributed:

$$A_{ij}^{\prime }=A_{ij}\times D_{i}$$

Thus, for cross-genome comparisons, we use the normalized attention scores $A_{ij}^{\prime }=\left\{\begin{array}{ccc} A_{i1} & \cdots & A_{in_{i}} \end{array}\right\}$. In our above example, ${A^{\prime }}_{i}=\left[\begin{array}{cc} 0.0 & 0.0 \end{array}\right]$ and ${A^{\prime }}_{k}=\left[\begin{array}{cccc} 0.0881 & 0.0528 & 0.0088 & 0.0264 \end{array}\right]$.

Then, when analyzing the association of vPST attention with protein function, we first clustered the proteins using sequence identity (mmseqs2 v13.45111 -e 1e-3 -c 0.5 -s 7.5). We computed the max scaled attention $A_{ij}^{\prime }$ for all proteins in the same cluster. For function association analyses, we additionally retained 50 protein clusters with the largest $A_{ij}^{\prime }$ values for each functional category curated in the VOG r219 and PHROG databases.

### Protein annotation improvement

We considered all proteins unable to be annotated using the VOG r219 or PHROG databases as hypothetical proteins, where $N_{H}$ is the number of hypothetical proteins. We computed the annotation improvement as a function of a genome clustering assignment and protein embedding. We used the same protein search settings described in the Methods section “Clustering genome and protein embeddings”: cosine similarity on the unit-normalized protein embeddings, restricted to proteins that belong to genomes in the same genome cluster. For each protein, we searched for the closest non-self protein, scoring this as an improvement if this neighbor protein was annotated:

$$T=\sum_{i=1}^{{\text{N}}_{\text{H}}}\left\{\begin{array}{cc} \text{if}\;{\text{NearestNeighbor}}_{\text{i}}\;\text{is}\;\text{annotated} & 1\\ \text{else} & 0 \end{array}\right.$$

Then, we computed the overall annotation improvement AP as the proportion of hypothetical proteins whose nearest neighbor was annotated:

$$AP=\frac{T}{N_{H}}$$

For a given genome embedding, we also computed the rate of change of AP over the number of nearest genome neighbors used for genome clustering using the numpy.polyfit (v1.23.5) function.

### Protein function co-clustering

We used curated PHROG functional categories (Supplementary Table 5) to compute functional co-clustering, excluding the category for proteins of unknown function. Given a genome clustering and protein clustering configuration, for each protein cluster $P_{i}\in P$, we count the co-occurrence between pairs of functional categories u and v as the product of the number of proteins belonging to each category in the cluster:

$$C_{i}^{uv}=n_{i}^{u}\times n_{i}^{v}$$

where $n_{i}^{u}$ is the number of proteins in the ith protein cluster that belongs to category u. The observed co-occurrence $C^{uv}$ between the functional categories u and v is defined as the sum of cluster-level co-occurrences:

$$C^{uv}=\sum_{i=1}^{\left|P\right|}C_{i}^{uv}$$

To account for the distribution of PHROG annotation profiles, we computed an enrichment score against the background of the distribution of the 38,800 PHROG profiles:

$$E^{uv}=\frac{C^{uv}}{C_{b\text{ackground}}^{uv}}\in \left[0,\infty \right]$$

where $C_{b\text{ackground}}^{uv}$ is computed analogously to $C^{uv}$ except using relative abundances of the annotation profiles themselves instead of annotated proteins. To identify functional categories that co-occur frequently, we constructed a fully-connected graph with all PHROG functional categories as nodes and the corresponding edge weights $E^{uv}$ between categories u and v. We then applied the Leiden algorithm with resolution 1.0 to identify sub-communities of co-occurring functions enriched above background.

### Protein functional module detection

We defined the following protein functional modules based on curated functional categories (Supplementary Tables 5 and 6) and annotation text searches. For replication proteins in the PHROG database, we included proteins that were initially categorized as “nucleotide metabolism” and had matches to the following regex pattern “(?i)DNA pol|single strand DNA binding|Par[AB]|DNA primase|(DNA)?[ ]?helicase|repl|primosom|terminal|ribonucleo[st]ide(.*)?reductase|NDP reductase”. For VOG, we considered all hits in the replication category. For PHROG packaging modules, we included hits that belong to the “head and packaging” category and specifically matched the regex pattern “(?i)terminase|portal”. For VOG, we only considered those in the “packaging” category. For PHROG DNA-interacting modules, we included all hits that belonged to either “nucleotide metabolism”, “lysogeny”, or “gene expression” categories. For VOG, all hits belonging to “replication”, “integration”, “packaging”, and “gene expression” were included. For PHROG late genes, annotations in the categories “tail”, “head and packaging”, “connector”, and “lysis” were retained. Likewise, for VOG, the categories “structural”, “exit”, and “packaging” were included.

We considered protein clusters to correspond to a specific functional module if they met the following module-specific criteria: For searches that only considered a single functional category (replication, packaging), we required at least 2 proteins from that category with different annotations. Due to the volume of data, we could not ensure that the 2 different annotations referred to truly different protein functions and not just the same function worded differently. For multi-category searches (late genes, DNA-interacting), we required at least 2 categories to be represented. In either case, we excluded protein clusters that had any annotated proteins outside the indicated functional categories to focus on protein clusters that most strongly fit our definition of functional modules.

### Capsid structure searches

To quantify the frequency at which embedding-based protein clusters co-cluster VOG-detectable capsid proteins (VOG bit score ≥ 75) with proteins unable to be assigned function by VOG, we excluded all embedding-based protein clusters that did not solely consist of annotated capsids and hypothetical proteins. We then filtered this candidate set of proteins to keep those that fit the previous criteria at least 10 times among all clustering configurations. We additionally include sequence identity-based clusters (mmseqs2 v13.45111 cluster -s 7.5 -c 0.5) that also consisted of unannotated and capsid proteins as positive controls. This led to a total of 100,704 proteins for this analysis.

We used foldseek^34^ (v9.427df8a) to convert our protein sequence database into a 3Di-structure database using the ProstT5^35^ model (downloaded July 2024; foldseek createdb with “—prostt5-model” option), which uses language tokens to represent structural features. We searched our 3Di-structure database against 295k structures from the Protein Data Bank^36^ (PDB; downloaded using foldseek in July 2024) using default settings. We excluded all alignments with bit scores less than 100 and manually annotated the PDB structures as viral capsids using the following query at the PDB web server (https://www.rcsb.org): “capsid, major capsid, coat, minor capsid, virion”. We validated this approach by aligning AlphaFold 3-modeled^38^ (https://alphafoldserver.com) monomer structures with the HK97 major capsid protein (2FS3) using TM-align^59^ implemented in the PDB web server. We choose 2 proteins with the highest scoring structural alignment as determined by foldseek, each from either proteins annotated with a VOG profile of unknown function or proteins undetected by VOG, for this analysis.

We then scored the proportion of unannotated proteins in each cluster that had a structural alignment with a PDB capsid protein. To summarize these proportions for each combination of clustering hyperparameters, genome embedding, and protein embedding, we computed a weighted average of these proportions, using the cluster size as the weight.

### Embedding UMAP visualization

We used the Python implementation of the UMAP algorithm^60^ (umap-learn v0.5.3) for embedding visualization only. For genome embeddings, we used 15 nearest neighbors defined using Euclidean distance. When computing the reduced embeddings, we jointly embed the genome embeddings of both the training and test datasets for each type of genome embedding into the same space. For protein embeddings, we first unit-normalized each protein embedding to have an L2-norm of 1. Then, we used 8 nearest neighbors defined using cosine distance as this value gave the best visual separation. In both cases, we did not reduce the dimensionality before visualization, so the embeddings themselves were directly used as inputs to UMAP algorithm.

### Graph-based host prediction framework

For the virus host prediction proof-of-concept, we modeled our framework off CHERRY^39^, which applies graph learning on a virus-host interaction network $G=\left(X,E\right)$. Our implementation uses PyTorch (v2.2.2), PyTorch-Geometric (v2.5.2) and PyTorch-Lightning (v2.2.4). The interaction network is bipartite, meaning that there are 2 types of nodes: viral nodes $v_{i}\in V$ and host nodes $h_{i}\in H$. The total node set X is thus X=V∪H. The edges E represent known virus-host pairs and may also include confident virus-host predictions that come from virus-host genome alignments (see Host prediction training and test datasets for more detail). Given G, the objective is a link prediction task to infer for any virus-host pair $\left(v_{i},h_{i}\right)$ the probability of an edge existing in the interaction network.

For the most comparable analyses, we designed our neural network architecture based on CHERRY: an encoder consisting of multiple Graph Convolution^61^ (GCN) layers and a decoder that performs the link prediction. The encoder propagates information in the genome embeddings among local neighborhoods. Specifically, the GCN encoder layers can mathematically be represented as:

(6) $$e^{\left(l+1\right)}=\phi \left({\tilde{D}}^{-\frac{1}{2}}\tilde{A}{\tilde{D}}^{\frac{1}{2}}e^{\left(l\right)}W^{\left(l\right)}\right)$$

where l is the layer index, $\tilde{A}$ is the adjacency matrix with self-connections ( $\tilde{A}=A+I$, I is the identity matrix, ), and $\tilde{D}$ is the diagonal matrix where ${\tilde{D}}_{ii}=\sum_{j}{\tilde{A}}_{ij}$. ϕ is the activation function, and $W^{\left(l\right)}$ is the l-layer model weights. $e^{\left(0\right)}\in {\mathbb{R}}^{\left|X\right|\times N}$ is the input genome embedding where N is the size of the embedding. To compare our work to CHERRY, which uses tetranucleotide frequency genome embeddings, we substitute the genome embedding with either the vPST genome embeddings or the simple average of the ESM2 protein embeddings over each genome for both the viruses and hosts.

The decoder is a 2-layer feedforward neural network that takes the outputs from the encoder as input. We consider all possible virus-host pairs $\left(v_{i},h_{i}\right)\in X$ as a query set Q where each pair is represented by the difference in encoder embedding:

$$q_{ij}=\text{encoder}\left(v_{i}\right)-\text{encoder}\left(h_{i}\right)$$

The decoder, therefore, is mathematically written as:

$$\left\{q_{ij}^{\left(l+1\right)}=\phi \left(q_{ij}^{\left(l\right)}\theta^{\left(l\right)}\right)\;\text{decoder}\left(q_{ij}\right)-\text{sigmoid}\left(q_{ij}^{L-1}\right)\right.$$

where $q_{ij}^{\left(l\right)}$ is the hidden feature in the lth layer out of L total decoder layers, and $q_{ij}^{\left(0\right)}=q_{ij}$. ϕ is the activation function, and $\theta^{\left(l\right)}$ represents the weights of the lth fully connected layer.

### Host prediction training and test datasets

For the virus host prediction proof-of-concept, we modeled our framework off CHERRY^39^, which applies graph learning on a virus-host interaction network. To construct the network of known virus-host pairs, we used the train and test datasets from iPHoP^40^. Specifically, the train dataset included 3628 complete bacterial and archaeal viruses from NCBI RefSeq prior to 2021. The iPHoP test dataset consisted of 1636 complete bacterial and archaeal viruses from NCBI GenBank, distinct from the training dataset. Although both datasets indicate the taxonomy of the host, they do not provide specific genome accessions to link the viruses, which are necessary to construct the interaction network.

For the training dataset, we used the Virus-Host Database^62^ (accessed April 2024) to determine the full host taxonomy. We then selected either the NCBI RefSeq representative sequence associated with the host taxonomy, if one existed, or the most complete (longest and assembly_level == “Complete Genome”) genome from NCBI GenBank (accessed May 2024). We included all hosts in the Virus-Host Database if there were multiple such as in the case of viruses with a relatively broad host range. The set of hosts notably includes multiple strains of the same species or species of the same genus as indicated in the Virus-Host Database. Then, any strain information was ignored, so the lowest level of evaluation was at the host species.

We performed a similar search for the test dataset using the information provided in Supplementary Table 2 of iPHoP^40^. We divided the test virus hosts whose species ranks were unknown (ie “Wolbachia sp.”) into 2 different sets. If these hosts were already in the set of hosts for the training dataset, we did not retrieve any new host genomes. Instead, we considered all hosts currently in the set of hosts with the same genus as potential hosts for these viruses. For new hosts, we used the same search criteria as above to add a single new host for each of these viruses. This resulted in a total of 805 host genomes, corresponding to 594 unique host species.

### Constructing the virus-host interaction network

To construct the virus-host interaction network, we constructed the heterogeneous graph G that has 2 node types (virus, host) and 2 edge types (virus-related to-virus, virus-infects-host). For the virus-host edges, we included all virus-host pairs identified above, meaning that G includes both training and test viruses. We notably deviated from the CHERRY implementation by excluding confident host predictions that would have come from virus-host BLASTn genome alignments (proviruses) or CRISPR spacers. This deviation is not concerning since we focused on the relative performance of our vPST genome embeddings compared to other tools and genome embeddings, rather than absolute predictive ability.

To select virus-virus edges representing pairs of similar viruses, we used a protein sharing network clustering approach when using tetranucleotide frequency genome vectors. We first excluded all singleton proteins since these do not inform about genome-genome relatedness and only serve to account for the proportion of gene sharing relative to the total number of proteins/protein clusters in each genome. After protein clustering using mmseqs2 (v13.45111 -s 7.5 -e 1e-3 -c 0.5) and filtering singleton proteins, we constructed a sparse $\left(\left|V\right|\times n_{pc}\right)$ presence-absence matrix where $n_{pc}$ is the total number of protein clusters in the dataset. Each row represents what protein clusters are encoded in the indicated genome. We then computed the dice similarity $S_{ij}$ for each pair of genomes as:

$$S_{ij}=\frac{2\left|v_{i}\cdot v_{j}\right|}{\left|v_{i}\right|+\left|v_{j}\right|}$$

where $v_{i}$ and $v_{j}$ are the row presence-absence vectors for the ith and jth genomes, respectively. We then constructed a graph with all viruses where the edges are $S_{ij}|S_{ij}>0$. To understand which viruses were considered related, we clustered this graph with the Leiden algorithm with a resolution of 0.1. Edges were created in the interaction graph between all viruses in the same gene-sharing clusters. For the other genome embeddings we tested, we considered pairs of viruses to be related if their genome embeddings were at least 90% similar based on a Gaussian-kernel of Euclidean distances (Equation 4). We then pruned these embedding-based virus-virus connections to only maintain the top 15 neighboring viruses for each virus.

### Host prediction model training

We trained new graph-based host predictions models using the iPHoP training dataset, swapping the genome representations for vPST genome embeddings or the simple average of the ESM2 protein embeddings over each genome. Our implementation used PyTorch (v2.1.2) and PyTorch-Geometric (v2.4.0). We used a binary cross entropy loss objective for the link prediction task to classify the edge $E_{ij}$ as existing (1) or not (0):

$$𝓛=-\frac{1}{N}\sum_{k=1}^{N}y_{k}\log\left(p\left(y_{k}\right)\right)+\left(1-y_{k}\right)\log\left(1-p\left(y_{k}\right)\right)$$

where $y_{k}$ is the discretized final output for the 𝑘*th* virus-host pair from the model decoder, given a probability threshold for whether an edge $E_{ij}$ is predicted to exist.

During training, we randomly split all virus-host edges $E=\left\{E^{\left(T\right)},E^{\left(V\right)}\right\}$ into disjoint training $E^{\left(T\right)}$ and validation $E^{\left(V\right)}$ sets at an 80:20 ratio. We additionally randomly sampled negative edges $E^{\prime }=\left\{{E^{\prime }}^{\left(T\right)},{E^{\prime }}^{\left(V\right)}\right\}$ that do not exist in the virus-host interaction network G to provide the model with negative examples (implemented by PyTorch-Geometric). The negative edge sets ${E^{\prime }}^{\left(T\right)}$ and ${E^{\prime }}^{\left(V\right)}$ are also disjoint, and $\left|E\right|=\left|E^{\prime }\right|$ so that there was not label imbalance. During the message-passing stage of the model encoder, only the real edges E are used. After message passing updates the node representations, we used $E\cup E^{\prime }$ for decoding and inference with both real and negative edges. Therefore, $N=\left|E^{\left(\cdot \right)}\cup {E^{\prime }}^{\left(\cdot \right)}\right|$ for either the training or validation edges. Since the prediction task does not depend on virus-virus edges, these edges were not split or negatively sampled. This means that the graph structure and message passing consider all viruses, not just training viruses. Thus, during training, we masked any virus-host edges that contain test viruses in the loss computation to prevent data leakage.

Although we strived to implement a nearly 1:1 model with the original CHERRY implementation, our implementation and training deviates in 3 ways. (1) We allowed separate learnable weights for each type of edge (virus-virus, virus-host, and host-virus) in the message-passing encoder layers by updating Equation 6:

$$W^{\left(l\right)}=\left\{\begin{array}{cc} W_{vv}^{\left(l\right)} & \text{virus-virus}\;\text{edges}\\ W_{vh}^{\left(l\right)} & \text{virus-host}\;\text{edges}\\ W_{hv}^{\left(l\right)} & \text{host-virus}\;\text{edges} \end{array}\right.$$

$W_{hv}^{\left(l\right)}$ and subsequently host-virus edges are required to ensure reciprocal message passing between virus and host nodes despite the intuitive way of representing virus-host edges as directed. The native CHERRY implementation does not allow for edge type-specific weights, instead sharing weights for all edge types.

(2) Due to modeling G as a heterogeneous graph, the message passing layer is not a true Graph Convolution (GCN) layer, which is not implemented for heterogeneous graphs in PyTorch-Geometric. Specifically, we use a generalization of the GCN layer^63^ that allows for heterogeneous graph learning with multiple node and edge types. For this layer, however, the node update equations for this layer and the GCN layer are identical, but there may be PyTorch-Geometric implementation-specific differences beyond changing node representations.

(3) We explored a more sophisticated technique for handling the training and validation splits for link-level tasks that we refer to as “disjoint training”. Specifically, we divided the real training edges $E^{\left(T\right)}$ into 2 disjoint sets $E^{\left(T\right)}=\left\{E^{\left(MP\right)},E^{\left(D\right)}\right\}$ where $E^{\left(MP\right)}$ are edges only used for message passing (node updates) and $E^{\left(D\right)}\cup {E^{\prime }}^{\left(T\right)}$ are edges only used for supervision (decoding and inference). Specifically, $E^{\left(D\right)}\cup {E^{\prime }}^{\left(T\right)}$ are the edges used for link prediction. We only considered a 70:30 split for $E^{\left(MP\right)}$ and $E^{\left(D\right)}$ for this study when this was enabled. This modification is analogous to splitting training data into separate training and validation sets to prevent data leakage but only for training edges.

To decouple the effect of these 3 differences from the choice of node embeddings, we trained a model that is nearly faithful to the CHERRY implementation without these changes (barring the required change #2), and then we trained a separate model using tetranucleotide frequency genome embeddings (kmer) that enables our changes. Thus, the CHERRY and “kmer” model use the same virus-host interaction graph as input, but the “kmer” models explored the effects of changes #1 and #3.

To lightly optimize hyperparameters, we sampled from sets of intuitive values for the number of encoder layers, decoder hidden dimensions, learning rate, whether to enable disjoint training (at a 70:30 split), and whether to allow edge specific-weights in the encoder or not. We did not dilate the input embedding dimension in the encoder layers. For the 2-layer feedforward decoder network, we only chose values smaller or equal to the input embedding dimension for the first layer. The second layer dimensions were then required to be strictly less than the first layer dimensions. See Supplementary Table 9 for the values sampled for each hyperparameter. We applied the same random seed when training each iteration and chose the best model based good overall performance and lowest validation loss at the end of 150 training epochs. We defined “good” overall performance as a validation loss curve that was monotonically decreasing over or constant at the end of training time. We selected a total of 4 models that were the best: CHERRY without the above changes and 3 that allowed the above implementation changes and used different genome embeddings. All models were trained with a dropout of 0.25 after the encoder and after each decoder feedforward layer. We used the ReLU activation function after each layer.

### Host prediction model evaluation

iPHoP (v1.3.3) and each of the 4 trained models were evaluated using the iPHoP test dataset (see “Host prediction training and test datasets”). For the 4 graph-based models, we considered all test virus-host pairs for link prediction and retained only those ≥75% confidence, which is the minimum for iPHoP, or ≥90% confidence. All virus-host pairs were considered to enable resolution at each host taxonomic rank. However, we only evaluated if the true host taxon was among the predictions above the confidence threshold, so not all predictions were analyzed. Specifically, we computed the proportion of the iPHoP test viruses whose true host taxon was confidently predicted.

Since there were notably a nontrivial number of viruses in the iPHoP test dataset that were similar to those in the vPST training dataset based on AAI (see “Average amino acid identity (AAI) genome clustering”), we filtered these viruses out using several similarity cutoffs to evaluate their effects on our interpretation of the host prediction results.

## Supplementary Material

Supplement 1

## Data Availability

Sources for publicly available viral genomes are listed in Supplementary Table 1. Supplementary data specific to this manuscript, including protein FASTA files, protein and genome embeddings, trained vPST model weights, and virus-host interaction graphs, were deposited at DRYAD: (doi: 10.5061/dryad.d7wm37q8w). The repository will be made public after the completion of our biosecurity review.
