# Supplementary material for: Protein Set Transformer: A protein-based genome language model to power high diversity viromics
Source: Res Sq. 2024 Sep 23:rs.3.rs-4844047. Preprint. [Version 1] doi: 10.21203/rs.3.rs-4844047/v1 (PMC11469463; doi:10.21203/rs.3.rs-4844047/v1)
Supplement: Supplement 1 [file NIHPPrs4844047v1-supplement-1.pdf]

Supplementary Files

This is a list of supplementary files associated with this preprint. Click to download.

- ExtendedDataFigures.docx
